# Long term effects of crop rotation and fertilization on crop yield stability in southeast China

**DOI:** 10.1038/s41598-022-17675-1

**Published:** 2022-08-20

**Authors:** Licheng Zhang, Jie Yuan, Mingqing Zhang, Yongchun Zhang, Limin Wang, Juan Li

**Affiliations:** 1 Soil and Fertilizer Institute of Fujian Academy of Agricultural Sciences, Fuzhou, 350013 Fujian Province People’s Republic of China; 2 Agricultural Resources and Environment Institute of Jiangsu Academy of Agricultural Sciences, Nanjing, 210014 Jiangsu Province People’s Republic of China

**Keywords:** Psychology, Ecology

## Abstract

The objective of this study was to determine the effects of different fertilizer treatments and crop rotations on crop yield stability. A 9 years field experiment was conducted from 2013 to 2021 to evaluate the effects of combinations of two annual crop rotations and two methods of applying fertilizer on crop yield stability. Crop rotations were kidney bean–mustard–rice (P–B–O) and kidney bean–mustard–cowpea (P–B–V) each year. Fertilization methods were recommended fertilization (RF) and conventional fertilization (CF). The indexes Wi^2^ (Wricke’s ecovalance), coefficient of variation (CV), and sustainable yield index (SYI) were used to quantify the long term effects of crop rotation and fertilization on crop yield stability, and a yield change trend model was used to predict future production. For fertilization program RF, average kidney bean and mustard yields in rotation P–B–O increased respectively by 7.47% and 19.37% over P–B–V in the 9 years of the project. For CF, average kidney bean and mustard yields for P–B–O increased respectively by 14.99% and 18.33% over P–B–V. Wi^2^ indexes of kidney bean and mustard for P–B–O (respectively 116 and 956) were significantly less than for P–B–V (respectively 147.87 and 1259.67). SYI for kidney beans and mustard in P–B–O (respectively 0.63 and 0.57) were significantly greater than for P–B–V (respectively 0.50 and 0.42). The trends of crop average yields for RF and CF show that the average yield trends of kidney bean in P–B–O (respectively 32.41 and 32.34) were greater than in P–B–V (respectively 29.56 and 27.45). The trends of average yields of mustard for RF and CF in P–B–O (respectively 64.18 and 60.87) were greater than in P–B–V (respectively 51.74 and 51.87). The preceding results led to the conclusion that long term annual P–B–O rotation combined with RF considerably increased yield and maintained yield stability, thus establishing the sustainability of this cropping system.

## Introduction

The key to developing sustainable agriculture is to produce high crop yields and to maintain yields using a method of fertilization that is ecologically friendly to the natural environment. In agricultural practice, excessive fertilization and an imbalance of N, P, and K in soil decrease the stability of crop yield^1^. A sustainable scientific fertilization and tillage system must be established to increase agricultural production capacity and thus crop yield and to maintain stable agricultural production. It is therefore important to understand the impact of different fertilization and tillage systems on crop yield stability and predict future yield. In conventional fertilization methods, farmers prefer to increase the application of nitrogen fertilizer because nitrogen fertilization improves soil fertility and increases crop productivity^2^. For example, continuous application of nitrogen (120 kg/ha) and phosphorus (52 kg/ha) fertilizers can increase maize yield by up to 200% over 32 years^3^. In the Yangtze River basin, the application rate of N fertilizer ranges from 200 to 300 kg/ha, although the rice grain yield is only 6.7–7.6 t/ha^4^. The effect of excessive fertilizer on crop yield is not well established, but it indeed leads to an imbalance of soil nutrients. Maintaining the balance of soil nutrients and increasing crop yield stability by optimizing fertilizer application is an important tillage management measure for the sustainability of the farmland ecosystem.

Analyses of yield stability have become more important in recent years since change in the soil environment is associated with decreased crop yield stability^5^. In the past, the analysis of yield stability has been largely confined to multi-environmental trials of crop cultivars and did not take into account the cropping system and interannual climate differences^6^. Analysis of crop yield stability shows year-to-year variability in the form of a year × treatment interaction effect, an indicator that provides more information than average yield. However, it is difficult to explain interactions using a conventional analysis of variance approach. Wi^2^ (Wricke’s ecovalance) and SYI (sustainable yield index) can be used to quantify the interaction between different treatments and different years^7^. Analyzing yield stability of intercropping systems is a difficult and complex task that requires field experiments that run over periods of years^8^. Long term experiments are invaluable for identifying slow changes caused by cropping systems that occur over the long term and they can reveal potential damage to the environment and threats to the future fertility of agricultural land^9^. A long term field experiment is subject to annual regional climate conditions and therefore provides a reliable basis for studying changes in soil fertility and crop yield and can support the development of a practical fertilization program. Long term experiments have other advantages over short term trials and have become a vital component of agricultural science^10^ because they provide excellent opportunities to study crop yield trends over time and to investigate the effects of fertilization under different tillage and cultivation practices on crop yield, soil nutrients and the natural agricultural environment^11^.

Some studies have investigated the effects of long term fertilization on crop yield. Li et al. showed that a combination of organic and inorganic fertilizer improved both yield and yield stability for winter wheat^12^. In a 33 years wheat–maize rotation experiment, Yang et al. found no immediate significant difference in yield between organic fertilizer combined with inorganic fertilizer and chemical fertilizer, but yield increased significantly over time due to previous fertilizer applications^13^.

Low soil fertility is a major constraint on agricultural productivity. Jin et al. found that the yield of rice crops in China increased year by year and that the contribution rate of fertilizers to the increase in rice yield was up to 50%^14^. Wang et al. used meta-analysis to determine the relationship between rice yield and fertilizer application in the main rice-producing areas in China and found that individually applying nitrogen, phosphorus or potassium fertilizers significantly increased yield. Compared with no fertilizer application, yield increases reached 35.1% (N), 10.9% (P) and 11.9% (K)^15^. The application of fertilizer is the conventional method of significantly increasing soil nutrients to promote crop production. However, imbalanced fertilization results in low fertilizer efficiency and has negative environmental effects, such as soil acidification, soil compaction and water eutrophication^16,17^. It is necessary to apply a balanced fertilizer to maintain crop yield stability and prevent environmental damage. Balancing fertilizer application depends on the basic soil fertility and crop nutrient demand. Good fertilizer management is a critical factor of crop yield because it increases the utilization of soil nutrients by plants and is therefore necessary to maximize agricultural production^18,19^. Mathematical models of crop fertility have been developed that depend on large quantities of field test data and they generally show that science-based fertilization methods increase the effectiveness of fertilizer application, decrease fertilizer waste and reduce the loss of nutrients^20–22^. Zhang et al. measured the fertility of vegetable soil in the hilly area of southern Fujian Province in China and developed a recommended fertilization program based on crop growth and nutrient demand^23^. To date, the quantities to be applied in most recommended fertilization programs have been determined from short term field productively indicators. However, some limited information pertaining to the long term effects of managed fertilizer application on crop productively and its sustainability in hilly area soils is available. Results from long term field experiments allow us to explore the mechanisms underlying the effects of fertilization on crop yield and soil quality. We therefore engaged in field monitoring and multiyear data collection in a long term fertilization experiment of vegetable–rice rotation in Fujian Province in China that commenced in 2013. Our objectives were: (1) to determine the effects of long term fertilization on yield stability in a vegetable–rice rotation system; and (2) to investigate the mechanisms underlying the changes in crop yield in a vegetable–rice rotation and the effects of fertilizer application. This study identifies changes in crop yield for vegetable–rice rotation and provides a scientific basis for improved crop fertilizer application and the maintenance and improvement of soil productivity.

Rice is the most widely planted food crop in Fujian Province, China, and green bean, mustard, and cowpea are the largest vegetable crops in the province^24^, with annual planting areas of 1000 to 2000 km^2^. These crops are thus very important in regional agricultural production. P–B–O and P–B–V rotations are important cropping systems in this region. Crop rotation is the alternate planting of different crops on the same cultivated land, and can include paddy and dry land rotation. Local farmers apply fertilizer to each crop in order to increase yield, which causes a serious excess of fertilizer. It is urgently necessary to reduce the excessive application of chemical fertilizer by optimizing fertilizer mixes and applications and promoting fertilizer savings and efficient fertilizer use to increase or maintain crop yields under local rotation practices. Zhang et al. created fertilization models for various locally grown crops, such as rice, kidney bean, mustard and cowpea, in Fujian Province through long term experimental research. The model calculates the recommended fertilizer amount according to the measured basic soil fertility data for the cultivated land^23^. The recommended fertilizer application program used in the field experiment clearly increased crop yield and produced economic benefits. However, there are few studies on the yield stability of P–B–O and P–B–V rotations using a recommended fertilization model. Adopting the recommended fertilizer application program can in practice, whether using the P–B–O rotation or the P–B–V rotation, maintain yield stability of rotated crops for a long period. Investigating this problem will enable us to provide a scientific basis for fertilizer application and ensure the long term sustainability of rotated crops. Our research findings will add to the body of knowledge gained from similar research and will serve as an effective tool to analyze trends, generate models and determine the most suitable crop production techniques to be used in order to reduce variation in yields and achieve sustainable production.

## Materials and methods

### Site description

**Figure 1:**
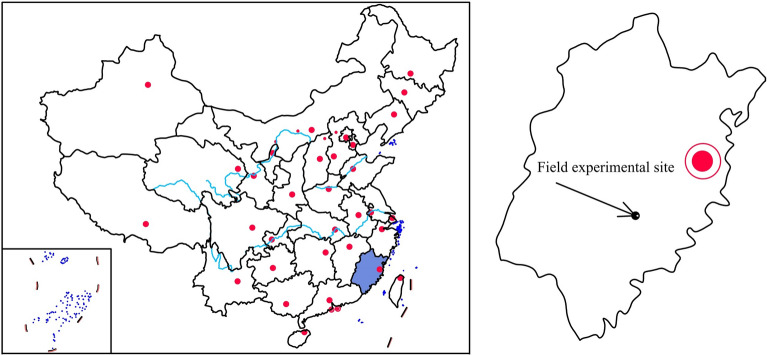
Location of the field experiment site.

The field experiment was initiated in 2013 at the Yongchun County, Fujian Province, China (25°12′37″ N, 118°10′24″ E), using the two rotations of vegetables and rice (Fig. 1). The site is in the north of the Tropic of Cancer, with a typical subtropical marine monsoon climate, sufficient sunshine, and average annual solar radiation 462.26 kJ/cm^2^. The climate is mild and humid, with average annual temperature 16–21 °C and average annual rainfall about 1400 mm. Agricultural production allows for the cultivation of three crops annually. The soil of the test field was lateritic red soil.

### Experiment design

The experiment was conducted over 9 years from 2013 to 2021. Soil samples were collected before the experiment began to determine the main physical and chemical properties of the soil in the test plot, which were: organic matter content 19.96 g/kg, total nitrogen 2.25 g/kg, total phosphorus 1.31 g/kg, total potassium 27.86 g/kg, alkaline hydrolyzable nitrogen 107.73 mg/kg, available phosphorus 60.35 mg/kg, available potassium 116 mg/kg and soil pH 5.54. The test site was a rectangular field, 26 m long and 9 m wide, divided into 15 test blocks, each 5 m long and 2.8 m wide. Cement ridges were used to separate the test blocks, and irrigation drainage ditches were set outside the blocks. A protective isolation strip 1 m wide was formed around the test site. The experiment included two crop rotations: (I) rotation P–B–O: P, kidney bean (*Phaseolus vulgaris* L.), B, mustard (*Brassica juncea* L.), O, rice (*Oryza sativa* L.); and II) rotation P–B–V: P, kidney bean (*P. vulgaris* L.), B, mustard (*B. juncea* L.), V, cowpea (*Vigna unguiculata* L.). Four fertilizer treatments were selected: (1) recommended fertilization (RF) used with rotation P–B–O; (2) recommended fertilization (RF) used with P–B–V; (3) conventional fertilization (CF) used with P–B–O; (4) conventional fertilization (CF) used with P–B–V. A randomized complete block experimental design with three replications was used in the field study. The fertilization amounts used for treatments RF and CF are shown in Table 1. Under the CF, the amount of fertilizer applied to crops in each season is determined according to the years of fertilization habits of local farmers. The fertilization amount of crops in each season under the RF was calculated according to the measured basic soil fertility combined with the fertilization model of previous studies. The fertilization amount of crops in each season under the CF in this study is obtained by investigating the local farmers. The data on the fertilization amount of crops in each season under the RF is cited from the research report of Zhang et al.^23^. Urea (N 46%) was the nitrogen fertilizer, calcium superphosphate (P_2_O_5_ 12%) was the phosphorus fertilizer, and potassium chloride (K_2_O 60%) was the potassium fertilizer. All phosphorus fertilizer applied to crops in each season was used as base fertilizer, and nitrogen and potassium fertilizer were applied separately as base fertilizer (40% of the total fertilization) and topdressing (60% of the total fertilization). The topdressing method was that nitrogen and potassium fertilizer for kidney bean and cowpea were applied twice, 30% of the fertilization amount each time; nitrogen and potassium fertilizer for mustard was applied three times, 20% of the fertilization amount each time; nitrogen fertilizer for rice was applied at two different growing stages, 50% of the fertilization amount at the tillering stage and 10% of the fertilization amount at the panicle stage; potassium fertilizer was applied once, using 60% of the fertilization amount. The first crop, kidney bean, was sown in early September and harvested in November. The second crop mustard, was sown in early December and harvested in February of the following year. The third crop, rice or cowpea, was sown in early April and harvested in July.

**Table 1:** Fertilization rate of each treatment in the long term crop rotation experiment (kg/hm^2^).

Fertilization	Rotation	Kidney bean			Mustard			Rice			Cowpea		
		N	P	K	N	P	K	N	P	K	N	P	K
RF	P–B–O	150	45	105	270	75	150	75	0	60	–	–	–
	P–B–V	150	45	105	270	75	150	–	–	–	150	45	105
CF	P–B–O	171	67.5	67.5	286.5	204	189	141	45	0	–	–	–
	P–B–V	171	67.5	67.5	286.5	204	189	–	–	–	201	135	135

N fertilizer is urea, P is calcium superphosphate, K is KCl; the fertilization amount in the table was calculated from the chemical content of the fertilizer.

### Data analysis and methods

Yield stability analysis was conducted for the 9 years period using three different approaches. First, the coefficient of variation (CV) was calculated to give a measure of the temporal variability of yield for each treatment:

1 $$CV=\frac{\upsigma }{Y}*100 {\%}$$

where *σ* is the standard deviation of average crop yield in each year, and *Y* is the average crop yield in each year. A low value of CV indicates little variation, which implies that interannual difference in crop yield in the experimental plot is small and the yield is relatively stable over the years of the experimental period.

A second yield stability indicator is the sustainable yield index (SYI), which is calculated by Singh et al.^25^:

2 $$SYI=\frac{\mathrm{Y}-\upsigma }{{Y}_{max}}$$

where *Y* is the average annual crop yield, *σ* is the standard deviation of the average annual crop yield, and *Y*_*Max*_ is the maximum annual crop yield. A high value of SYI indicates a greater capacity of the soil to sustain a particular crop yield over time.

The third stability measure is Wricke’s ecovalence index (Wi^2^), which was calculated individually for each crop management system by Wricke^26^:

3 $${Wi}^{2}={\sum }_{j=1}^{q}({x}_{ij}-{{m}_{i}-{m}_{j}+m)}^{2}$$

where *x*_*ij*_ is the yield for treatment *i* in year *j*, *m*_*i*_ is the yield for treatment *i* across all years, *m*_*j*_ is the yield for year *j* across all treatments, and *m* is the average yield for all treatments across all years. When Wi^2^ is close to 0, the yield for treatment *i* is very stable.

### Analysis of crop yield trends

A simple linear regression analysis of grain yield (slopes and *P* values) over the years was performed to identify the yield trend (Choudhary et al.^27^):

4 $$Y=a+bt$$

where *Y* is the crop yield (t/ha), *a* is a constant, *t* is the time in years, and *b* is the slope, or magnitude of the yield trend (annual rate of change in yield).

Analysis of variance (ANOVA) was performed using MATLAB R2019b in order to compare crop yields in the long term experiment. Yield stability and univariate linear regression equations were created and statistically analyzed using the software toolbox. The coefficients of variation for yields, yield sustainability indexes, and graphs presented in this paper were calculated and drawn using MATLAB; differences were considered to be significant when *P* < 0.05.

### Interaction effects on crops yield

The long term crop yield data from this study provide a unique opportunity to assess yield and yield stability. Crops yield was influenced by the interaction of rotation tillage and fertilizer application, and environmental conditions (interannual change). To assess the complex interaction effect of factors on crop yield, analysis of variance (ANOVA) was performed for interaction effects that included interannual change × crop rotation, interannual variation × fertilization, crop rotation crop × fertilization, and interannual change × crop rotation × fertilization.

### Ethics approval

The experimental research and field studies on plants, including the collection of plant material, complied with relevant institutional, national, and international guidelines and legislation. The appropriate permissions and/or licenses for collection of plant or seed specimens were obtained for the study.

## Results and analysis

### Dynamic changes of crop yield from 2013 to 2021

The crop yield for each treatment varied greatly over the experimental period as crops were affected by climate, precipitation and other environmental factors. Yield changes of kidney bean, mustard, rice and cowpea crops for different treatments are shown in Tables 2 and 3.

**Table 2:** Changes in kidney bean and mustard yield in different years by rotation system for two fertilization modes (kg/plot).

Fertilization	Rotation	Kidney bean	Mustard	Fertilization	Rotation
		2013–2020 average	CV	2014–2021 average	CV
RF	P–B–O	32.95 ± 2.01a	6.20 ± 2.91b	55.34 ± 2.37a	4.29 ± 0.27d
	P–B–V	30.66 ± 2.75b	9.24 ± 5.59a	46.35 ± 2.75b	6.00 ± 1.83c
CF	P–B–O	33.07 ± 1.72a	5.16 ± 0.96c	54.29 ± 7.04a	13.17 ± 5.04b
	P–B–V	28.75 ± 2.38c	8.53 ± 4.79a	45.88 ± 6.85b	17.46 ± 2.19a

Numbers followed by the different letter in a column show significant difference at P < 0.05.

**Table 3:** Changes in rice and cowpea yield in different years by rotation system for two fertilization modes (kg/plot).

Fertilization	Rotation	Rice	Cowpea	Fertilization	Rotation
		2014–2021 average	CV	2014–2021 average	CV
RF	P–B–O	26.09 ± 2.67a	10.39 ± 10.97b	–	–
	P–B–V	–	–	44.19 ± 6.86a	15.84 ± 13.79b
CF	P–B–O	24.77 ± 2.99b	12.10 ± 11.47a	–	–
	P–B–V	–	–	41.92 ± 5.43b	12.94 ± 7.52a

Numbers followed by the different letter in a column show significant difference at P < 0.05.

For both RF and CF, mustard yield was significantly greater in P–B–O than in P–B–V. In both P–B–O and P–B–V, the kidney bean, mustard and rice/cowpea yields were significantly greater for RF than for CF. The average yields of crops in each season of the experiment over the 9 years period were as follows. For RF, the average yields of kidney bean and mustard in P–B–O were respectively 32.95 and 55.34 kg/plot; these values were 7.47% and 19.37% greater than in P–B–V. The average yields of kidney bean and mustard in P–B–O were respectively 33.06 and 54.29 kg/plot; these values were 14.99% and 18.33% greater than in P–B–V.

We also compared the coefficients of variation in crop yield for each treatment. CVs for kidney bean and mustard in P–B–O were significantly less than in P–B–V for both RF and CF. CVs for mustard, rice and cowpea were significantly less for RF than for CF.

### Effects of interactions on crops yield in 2013–2021

The experimental results for 9 consecutive years (Tables 2 and 3) show that crop yields varied significantly over seasons between fertilizer treatments. These results indicate that crop yield was affected by interannual change in climate and environmental factors, crop rotation, and fertilization programs. Interannual change, crop rotation and fertilization affected kidney bean and mustard yield, and interannual change and fertilization affected rice and cowpea yield. Three-way ANOVA (Table 4) showed that interannual variation had an extremely significant effect on kidney bean (F = 13.832) and mustard (F = 45.767) yield. Crop rotation had an extremely significant effect on kidney bean (F = 19.998) and mustard (F = 39.627) yield. Fertilization had a significant effect on kidney bean (F = 2.335) and mustard (F = 4.752) yield. Two-way ANOVA (Table 5) showed that interannual variation had an extremely significant effect on rice (F = 9.461) and cowpea (F = 50.047) yield. Fertilization had a significant effect on rice (F = 2.413) and cowpea (F = 3.496) yield.

**Table 4:** ANOVA for kidney bean and mustard under different rotation–fertilization treatments 2013–2020. Stars indicated the level of signifcance (*p < 0.05,**p < 0.01).

Variances	Kidney bean yield			Mustard yield		
	df	F	p	df	F	p
Interannual variation (I)	7	13.832**	0.000	7	45.767**	0.000
Crop rotation (C)	1	19.998**	0.000	1	39.627**	0.000
Fertilization (F)	1	4.752*	0.033	1	2.335*	0.035
I × C	7	1.423	0.238	7	3.432*	0.004
I × F	7	0.874	0.532	7	1.273	0.278
C × F	1	0.025	1.000	1	1.969	0.073
I × C × F	7	0.321	0.942	7	1.072	0.391

**Table 5:** ANOVA for rice and cowpea yield under different fertilization treatments 2014–2021. Stars indicated the level of signifcance (*p < 0.05,**p < 0.01).

Variances	Rice yield			Cowpea yield		
	df	F	p	df	F	p
Interannual variation (I)	7	9.461	0**	7	50.047	0.000**
Fertilization (F)	1	2.413	0.042*	1	3.496	0.007*
I × F	7	0.382	0.905	7	0.205	0.982

### Effects of fertilization and crop rotation on yield stability and crop sustainability

The stability index (Wi^2^) and sustainability index (SYI) of crop yield for each treatment are shown in Table 6. The yield stability indexes of kidney bean (116.06) and mustard (956.11) in P–B–O were significantly less than in P–B–V (respectively 147.87 and 1259.67). There was no significant difference in yield stability index between RF and CF. The yield stability indexes of rice and cowpea for RF were 308.30 (P–B–O) and 643.23 (P–B–V), which were significantly less than for CF (respectively 338.87 and 760.31). For RF, the yield sustainability indexes of kidney bean (0.63) and mustard (0.57) in P–B–O were significantly greater than in P–B–V (respectively 0.50 and 0.42). For CF, the yield sustainability indexes of kidney bean (0.60) and mustard (0.51) in P–B–O were significantly greater than in P–B–V (respectively 0.47 and 0.41). The sustainability indexes of crop yields for RF were greater than for CF. The sustainability index of rice yield for RF was 0.87 in P–B–O, which was significantly greater than the corresponding value for CF (0.77). The sustainability index of cowpea yield for RF was 0.55 in P–B–V, which was not significantly different to the value for CF (0.52).

**Table 6:** Crop stability indexes and sustainability indexes for different treatments. Different lowercase letters indicate signifcant difference between treatments (P < 0.05).

Fertilization	Rotation	Kidney bean		Mustard		Rice		Cowpea	
		Wi^2^	SYI	Wi^2^	SYI	Wi^2^	SYI	Wi^2^	SYI
RF	P–B–O	116.06 ± 87.50b	0.63 ± 0.04a	956.11 ± 436.54b	0.57 ± 0.04a	308.30 ± 115.45b	0.87 ± 0.05a	–	–
	P–B–V	147.87 ± 99.43a	0.50 ± 0.01b	1259.67 ± 811.03a	0.42 ± 0.02c	–	–	643.23 ± 228.91b	0.55 ± 0.04a
CF	P–B–O	124.04 ± 111.80b	0.60 ± 0.04a	1012.14 ± 488.44b	0.51 ± 0.05b	338.87 ± 110.74a	0.77 ± 0.04b	–	–
	P–B–V	157.86 ± 82.00a	0.47 ± 0.01b	1232.73 ± 434.29a	0.41 ± 0.02c	–	–	760.31 ± 293.94a	0.52 ± 0.03a

### Effects of fertilization and crop rotation on crop yield trends

**Figure 2:**
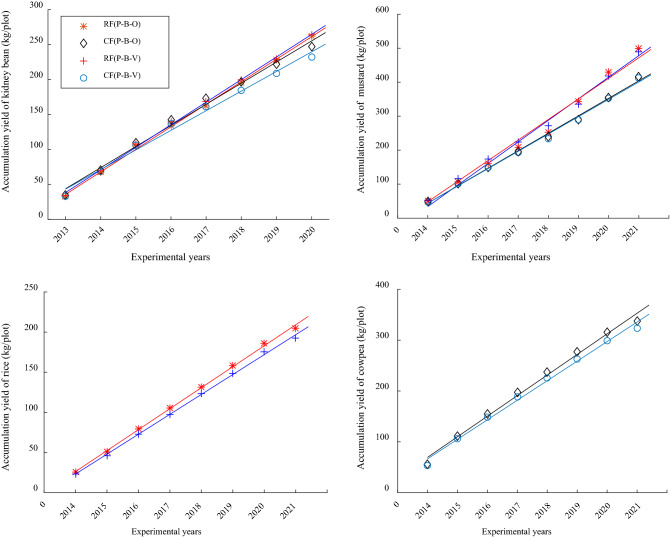
Change trends of annual cumulative yields of crops for different treatments.

A long term experiment provides important indexes that allow us to evaluate interannual change and identify future trends in crop yields. Crop yield is affected by many uncontrollable factors, which makes it difficult to obtain quantitative indexes that are statistically significant using yields that are predicted by trend-fitting regression analysis. However, cumulative crop yield for a particular treatment was recorded year by year and, using a grey system theory model, the two-dimensional scatter diagrams were drawn with cumulative yield as the ordinate and test time (years) as the abscissa. We concluded that there were linear relationships between crop yield and time (Fig. 2).

Crop yields were analyzed using a univariate linear model [Eq. (4)]. The results are shown in Tables 7 and 8. Regression analysis gave F values of crop yields for different treatments that indicated extremely significant differences. The goodness-of-fit index (R^2^), which indicates the strength of the relationship between yield and time, reached > 99%, suggesting that the fits were excellent. The trend of the mean value of crop yield for each treatment was fitted by the univariate linear model using the slope coefficient *b*. The trend (*b*) of kidney bean yield was P–B–O (32.41) > P–B–V (29.56) for RF, and P–B–O (32.34) > P–B–V (27.45) for CF. The trend of mustard yield was P–B–O (64.18) > P–B–V (51.74) for RF, and P–B–O (60.87) > P–B–V (51.87) for CF. The trends of the mean values of the yields of these two crops in P–B–O were greater than in P–B–V. The trend of the mean values of the yields of rice (the third crop) in P–B–O was 26.13 for RF, which was greater than for CF (24.81). The trend of the mean values of yields of cowpea (the third crop) in P–B–V was 40.51 for RF, which was greater than for CF (38.57). The confidence intervals of *b* were calculated using a grey model to assess the reliability of the trends of crop yields. The confidence intervals of *b* for kidney bean were 31.65–33.17 in P–B–O and 27.32–31.79 in P–B–V for RF, and 31.40–33.49 in P–B–O and 25.23–29.66 in P–B–V for CF. The confidence intervals of *b* for mustard were 58.30–70.05 in P–B–O and 49.11–54.38 in P–B–V for RF, and 56.96–64.38 in P–B–O and 48.92–54.84 in P–B–V for CF. The trends of the mean values of the yields of kidney bean and mustard do not overlap the confidence intervals of *b* in either P–B–O or P–B–V, which indicates that the trends of the yields of these two crops in P–B–O were significantly greater than in P–B–V.

**Table 7:** Linear grey model of yield trends of kidney bean and mustard for different treatments.

Fertilization	Rotation	Kidney bean				Mustard			
		*y* = *a* + *bt*	F Value	R^2^	95% confidence interval of *b*	*y* = *a* + *bt*	F Value	R^2^	95% confidence interval of *b*
RF	P–B–O	*y* = 2.99 + 32.41*t*	1017.10	1.00	31.65–33.17	*y* = − 31.73 + 64.18*t*	666.37	0.99	58.30–70.05
	P–B–V	*y* = 15.84 + 29.56*t*	974.80	0.99	27.32–31.79	*y* = − 8.42 + 51.74*t*	2150.2	1.00	49.11–54.38
CF	P–B–O	*y* = 6.11 + 32.34*t*	655.77	1.00	31.40–33.29	*y* = − 12.74 + 60.67*t*	1496.8	1.00	56.96–64.38
	P–B–V	*y* = 17.28 + 27.45*t*	856.83	0.99	25.23–29.66	*y* = − 10.19 + 51.87*t*	1719.7	1.00	48.92–54.84

**Table 8:** Linear grey model of yield trends of rice and cowpea for different treatments.

Fertilization	Rotation	Rice				Cowpea			
		*y* = *a* + *bt*	F value	R^2^	95% confidence interval of *b*	*y* = *a* + *bt*	F value	R^2^	95% confidence interval of *b*
RF	P–B–O	*y* = 0.12 + 26.13*t*	499.48	1.00	25.23–27.04	–	–	–	–
	P–B–V	–	–	–	–	*y* = 28.97 + 40.51*t*	722.71	0.99	36.82–44.19
CF	P–B–O	*y* = − 1.64 + 24.81*t*	514.41	1.00	23.96–25.65	–	–	–	–
	P–B–V	–	–	–	–	*y* = 28.00 + 38.47*t*	846.62	0.99	35.24–41.71

## Discussion

### Effects of rotation and fertilization on crop yields

Our results demonstrated that over the 9 years period of the experiment (2013–2021), yields in the P–B–O rotation were clearly better than those in P–B–V. In comparing the two crop rotations, a number of observations were made. The yields of the first and second crops (kidney bean and mustard) in P–B–O were greater than those in P–B–V. Average yields of kidney bean (32.95 kg/plot) and mustard (55.34 kg/plot) in P–B–O were respectively 7.47% and 19.40% greater than in P–B–O. P–B–O generally decreased annual variations in yields compared to P–B–V. In previous studies, crop rotation has an obvious effect of increasing crop yield. Sárvári and Pepó found that the mean yield of maize in rotation with winter wheat was 28% higher than monoculture maize^28^. Relevant research point out that crop rotation allowed for a reduction in fertilizer application and identified optimal fertilizer applications to chernozem soil^29^. In most cases, rotation results in greater soil porosity and increases nutrient availability for plant growth^30^. Our results are consistent with the results of previous studies that have shown that the paddy–upland rotation system increases yield^31^. Paddy–upland rotation improves the physical and chemical properties of soil, increases the availability of soil fertilizer, and thus increases crop yield^32^. Different crop rotations improve on continuous monoculture in various ways because of differences in nutrient requirements, reduced loss of fertilizer elements, and increased resistance to pests and pathogens. These advantages of crop rotation change yields. We found that the coefficients of variation for kidney bean and mustard in P–B–O were significantly less than in P–B–V. This shows that in the long term, P–B–O increased the capacity of the soil to supply nutrients and maintain crop yield stability. The effects of crop rotation on crop yields are also related to the application of fertilizer and field management practices^33^. Crop rotation combined with planned fertilization increases the amounts of mineralizable N, soil organic content, and aggregates in comparison with monoculture practices^34^. The magnitude of rotation effects decreased as N fertilization increased, a phenomenon that is often observed in legume–cereal rotations^35,36^. Although the main advantage of rotation and fertilization were manifest in crop yields, our results showed that there were significant differences in coefficients of variation for the third crop yields between RF and CF. In P–B–O, CV for rice yield was significantly less for RF than for CF. In P–B–V, CV for cowpea yield was significantly less for RF than for CF. The differences in the CV of crop yield may be due to the interaction between crop rotation and fertilizer application. P–B–V combined with RF had a significant effect on the yield in the third season, however a large varation in yield can easily be caused, so the CV was less than for CF. It is important to quantitatively index the interaction effects of crop rotation, fertilizer application and interannual change in order to evaluate the benefits to crop yield. Multivariate analysis of variance indicated that there were interactions between factors, but interannual change and rotation were the main factors that influenced crop yield.

### Yield stability and sustainability of long term rotation and fertilization

Long term experiments investigating crop yield are affected by temperature, moisture, crop variety, fertilization, management practices and other factors. The considerable year-to-year variability observed might be due to the interaction of year-specific climate or weather events and C input^37^. Our long term field experiment used only the parameters average yield and coefficient of variation, which are inadequate for a comprehensive evaluation of yield stability because environmental factors such as rainfall, temperature and solar radiation vary greatly interannually. As a crop grows, the interactions between environmental factors, fertilization and the rotation system result in interannual change in yield. We therefore used the stability evaluation index (Wi^2^) and sustainable yield index (SYI) to evaluate crop yield stability for different fertilizer–rotation treatments. SYI can take any value from 0 to 1. When the standard deviation is high, SYI will be close to 0, which suggests an unstable crop yield for the treatment concerned^38^. We found significant differences in Wi^2^ and SYI between different treatments for kidney bean and mustard. The yields of kidney bean and mustard in P–B–O under RF were more sustainable than for other rotation–fertilizer treatments. SYI of kidney bean was greatest in P–B–O, and Wi^2^ was least for RF. These results are consistent with the findings of Wang et al. that long term balanced application of N, P and K significantly increased crop yield and resulted in stable farmland productivity^39^. Change in crop yield over the period of our experiment showed that balanced fertilization significantly increased SYI and decreased the interannual coefficient of variation. In terms of yield stability and sustainability for different fertilization treatments, long term CV for crop yield was greatest and SYI was least for CF. This indicates that crop yield varied drastically, which in turn indicated that yield stability and sustainability were affected by fertilization. Using SYI to evaluate the effects of farming practices on crop yield stability is a valuable point of reference. Optimizing fertilization to increase SYI is important in maintaining the stability of agricultural production.

### The mechanics of rotation and fertilization maintain crop yield stability

Planting dryland crops after planting rice increases the yield of dryland crops. This may be because the change in soil from a flooded environment to dry land increases the availability of soil nutrients. In addition, residual rice roots decompose into soil organic matter, which promotes the release of soil nutrients and is an important factor in increasing the yield of dryland crops. Under conditions of alternating flood and drought, soil flooding during the rice season decreases soil permeability, thereby decreasing the leakage loss of nitrogen and phosphorus nutrients and increasing soil nutrient storage capacity. Alternate flooding and drought, affects soil moisture and alters the gas content of the soil. These changes accelerate biochemical reactions in the soil and act to balance soil nutrient supply. Our yield analysis for two different rotation methods showed that P–B–V produced a high yield under conventional fertilization. This was because crop yield is affected by basic soil nutrients, nutrient availability, and external inputs of nitrogen, phosphorus, and potassium fertilizers. The great variability of crop yield under conventional fertilization may be caused by the excessive external input of nitrogen, phosphorus, and potassium fertilizers.

## Conclusion

Crop yields for combinations of different rotations and fertilizer treatments in a 9 years field experiment showed that, in contrast to other treatments, the treatment of recommended fertilization in a P–B–O rotation produced the least interannual change in crop yield, increased yield stability, and resulted in a high yield as predicted by the trend model.

## Data Availability

The datasets generated and/or analysed during the current study are not publicly available due (These data collected from a fixed long term experiment, and restrictions apply to the availability of the data, which will be used to continue study in the future) but are available from the corresponding author on reasonable request.
